# Regenerative Performance and Structural Persistence of Silk Fibroin Matrices in Human Infected and Non-Infected Ex Vivo Wounds

**DOI:** 10.3390/biomimetics11080563

**Published:** 2026-08-06

**Authors:** Sophie C. Liegenfeld, Niklas P. Straub, Nicolas Krueger, Mandy Dittmer, Arianna Delle Coste, Jan T. Strenge, Markus Geissen, Sophie C. Rhode, Wolfgang R. Streit, Ralf Smeets, Ewa K. Stuermer

**Affiliations:** 1 Department for Vascular Medicine, Translational Wound Research, University Medical Center Hamburg-Eppendorf, 20246 Hamburg, Germany; niklas.straub@stud.uke.uni-hamburg.de (N.P.S.); ni.krueger@uke.de (N.K.); m.dittmer@uke.de (M.D.); m.geissen@uke.de (M.G.); e.stuermer@uke.de (E.K.S.); 2 Department of Oral and Maxillofacial Surgery, University Medical Center Hamburg-Eppendorf, 20246 Hamburg, Germany; a.dellecoste@uke.de (A.D.C.); j.strenge@uke.de (J.T.S.); r.smeets@uke.de (R.S.); 3 Department of Plastic, Reconstructive and Aesthetic Surgery, University Medical Center Hamburg-Eppendorf, 20246 Hamburg, Germany; s.rhode@uke.de; 4 Department of Microbiology and Biotechnology, University of Hamburg, 20148 Hamburg, Germany; wolfgang.streit@uni-hamburg.de; 5 Department of Oral and Maxillofacial Surgery, Division of “Regenerative Orofacial Medicine”, University Medical Center Hamburg-Eppendorf, 20246 Hamburg, Germany

**Keywords:** silk fibroin, wound healing, biofilm infection, biomaterial degradation, ex vivo skin model

## Abstract

Biodegradable biomaterials are promising candidates for regenerative wound care, yet their performance under infection-driven conditions remains poorly understood. This study evaluated the regenerative efficacy and structural persistence of a silk fibroin membrane and electrospun nonwoven matrix using a human ex vivo full-thickness skin wound model under non-infected and bacterially infected conditions. Complementary in vitro degradation assays assessed matrix durability following exposure to *Staphylococcus aureus*, *Pseudomonas aeruginosa*, bacterial culture supernatants and clinically relevant antiseptic solutions. In non-infected wound conditions, both matrices enhanced wound regeneration, resulting in increased re-epithelialization and proliferative activity compared with untreated controls; membrane-treated wounds achieved approximately 95% re-epithelialization after 15 days compared with approximately 20% in untreated controls, indicating accelerated wound closure, whereas nonwoven matrices supported sustained cellular proliferation over time. In contrast, bacterial infection was associated with almost complete absence of re-epithelialization and proliferative activity irrespective of matrix architecture. Matrix persistence was pathogen-dependent. While *S. aureus* induced moderate degradation, *P. aeruginosa* caused pronounced structural deterioration in ex vivo and in vitro models. Exposure to *P. aeruginosa* culture supernatants produced similar effects. These findings demonstrate that regenerative efficacy and material persistence are distinct biomaterial properties that are strongly influenced by the wound microbiological environment and are profoundly compromised under conditions of high bacterial burden.

## 1. Introduction

Wound healing is a highly coordinated biological process whose outcome is critically determined by the local wound environment [1]. While acute wounds generally progress through temporally orchestrated phases of inflammation, proliferation and remodeling, infected wounds are frequently characterized by persistent inflammation, elevated proteolytic activity, impaired re-epithelialization and the formation of bacterial biofilms, resulting in delayed or incomplete tissue repair [2,3]. Among the most prevalent pathogens associated with infected and chronic wounds are *Staphylococcus aureus* (*S. aureus*) and *Pseudomonas aeruginosa* (*P. aeruginosa*), both of which are strongly linked to biofilm formation, antimicrobial tolerance and the persistence of chronic wounds [4]. Infected wounds further exhibit excessive exudation, tissue degradation and dysregulated extracellular matrix turnover, creating a microenvironment that promotes microbial persistence against regenerative tissue repair [5,6]. These infection-driven wound environments also create proteolytic and inflammatory conditions that may fundamentally alter the structural integrity and biological performance of regenerative biomaterials [7].

Current wound dressings often provide passive protection and moisture control rather than actively supporting tissue integration and re-epithelialization, particularly in chronic or infection-associated wound environments [8]. Although acute wounds with low pathogen load generally retain regenerative capacity, extensive tissue defects and impaired wound coverage can still present substantial clinical challenges [9]. Therefore, advanced wound materials must balance structural versatility, biocompatibility and regenerative support while remaining stable within biologically compromised (chronic) wound environments to promote effective tissue regeneration across diverse wound conditions.

Silk fibroin has emerged as a promising biomaterial for regenerative wound applications due to its high biocompatibility, tunable mechanical properties and versatility in material fabrication [10]. Consequently, silk fibroin has been investigated in various forms, including films, hydrogels, electrospun membranes and nonwoven matrices for wound healing and tissue regeneration [11,12]. Beyond its structural versatility, silk fibroin actively supports several biological processes involved in tissue regeneration and wound healing. Silk fibroin-based matrices promote fibroblast and keratinocyte attachment, proliferation, migration, extracellular matrix production and re-epithelialization, while also serving as structurally supportive scaffolds for tissue integration and remodeling [13,14,15]. Despite their promising regenerative properties, most investigations of silk fibroin biomaterials have focused on sterile in vitro systems, material optimization strategies or antimicrobial functionalization approaches. Comparatively few studies have examined the interactions between regenerative matrices and infection-driven wound microenvironments in human tissue-based models [16,17,18]. In particular, little is currently known about how infection-driven wound environments influence tissue regeneration, matrix integration and the structural persistence of different silk fibroin architectures in clinically relevant human wound environments. Given the pronounced proteolytic activity and dysregulated extracellular matrix turnover associated with infected wounds, understanding the behavior of regenerative biomaterials under microbial burden is critical for their successful translational application in wound therapy [19,20].

In addition to host-derived proteases, both *S. aureus* and *P. aeruginosa* secrete extracellular enzymes, including proteases, capable of degrading structural proteins and extracellular matrix components, suggesting that infection-associated wound environments may directly affect the persistence and functionality of silk fibroin biomaterials [21,22]. Matrix architecture may further influence regenerative performance and material persistence in infected wounds. Differences in porosity, fiber organization, surface area and permeability may affect cellular infiltration, tissue integration, bacterial colonization and susceptibility to proteolytic degradation [23,24]. In particular, dense cast membranes and highly porous electrospun nonwoven matrices may interact differently with wound exudate, microbial biofilms and host tissue during regeneration, potentially resulting in distinct patterns of matrix stability and tissue remodeling [25,26]. Because successful wound biomaterials must simultaneously support tissue regeneration and maintain structural integrity within hostile wound environments—at least until a stable wound closure is achieved-regenerative performance and matrix persistence may represent distinct determinants of biomaterial function. However, these properties are rarely evaluated simultaneously, and it remains unclear whether biomaterials that perform favorably under regenerative conditions also retain structural functionality in infection-driven wound environments.

To investigate the interaction of regenerative biomaterials with infection-driven wound environments under physiologically relevant conditions, experimental models that preserve native human tissue architecture are required [27]. Human ex vivo skin wound models maintain the structural and cellular complexity of human skin while enabling controlled investigation of spatial pathogen–tissue–material interactions within a clinically relevant microenvironment [27,28]. As such, ex vivo wound systems provide an important translational bridge between reductionist in vitro approaches and animal wound models for studying biomaterial integration and wound healing under infected conditions. In this study, we compared two structurally distinct silk fibroin matrices, cast membranes and electrospun nonwoven matrices, in non-infected and infection-driven wound environments using a human ex vivo skin wound model. While previous studies have demonstrated the regenerative potential of silk fibroin-based wound matrices under non-infected conditions, the influence of infection-driven wound environments on matrix persistence and regenerative performance remains poorly understood. In particular, pathogen-dependent effects on biomaterial stability have not been systematically investigated in human tissue-based wound models. The study aimed to evaluate matrix-associated re-epithelialization, tissue integration and structural persistence under bacterial challenge with *S. aureus* and *P. aeruginosa*. In parallel, in vitro durability assays were performed to investigate matrix stability during exposure to clinically relevant antiseptic solutions and planktonic bacterial cultures. By distinguishing regenerative performance from intrinsic material persistence and by assessing pathogen-specific effects on matrix degradation, the study sought to define the context-dependent behavior and translational limitations of silk fibroin wound matrices under clinically relevant wound conditions.

## 2. Materials and Methods

### 2.1. Silk Fibroin Matrices

Medical-grade aqueous silk fibroin solution derived from *Bombyx mori* cocoons was provided by Fibrothelium GmbH (Aachen, Germany) using the proprietary PureSilk^®^ manufacturing process. Briefly, sericin was removed by degumming prior to fibroin dissolution in a non-toxic solvent system based on Ajisawa’s reagent. Silk fibroin membranes were generated by casting fibroin solution into polytetrafluoroethylene (PTFE) molds followed by drying for 24 h at room temperature under sterile laminar flow conditions, yielding a matrix thickness of approximately 0.05–0.1 mm. Additive-free silk fibroin nonwoven matrices were fabricated by electrospinning, followed by ethanol post-treatment to induce water insolubility and β-sheet stabilization, yielding matrices approximately 0.1–0.2 mm thick. Matrices (Fibrothelium GmbH, Aachen, Germany) were supplied sterile by the manufacturer and stored dry at room temperature until use.

### 2.2. Human Ex Vivo Skin Wound Model

Fully anonymized skin samples from three independent donors were obtained from patients undergoing elective surgical procedures at the Department of Plastic, Reconstructive and Aesthetic Surgery, University Medical Center Hamburg-Eppendorf, after written informed consent. Skin samples were processed immediately after surgical excision. Human full-thickness skin wound models were generated by preparing standardized 8 mm skin explants with a central 3 mm excisional wound using sterile biopsy punches as described previously [15]. Explants were placed epidermis-side up on sterile metal grids and cultured at an air–liquid interface in supplemented Dulbecco’s Modified Eagle Medium (DMEM; Gibco, Thermo Fisher Scientific; Waltham, MA, USA) containing 10% fetal bovine serum (Gibco, Thermo Fisher Scientific; Waltham, MA, USA). Cultures were maintained in a humidified incubator at 37 °C and 5% CO_2_ for up to 15 days, with culture medium replaced every 2 days.

### 2.3. Matrix Implantation into Wounds

Silk fibroin matrices were cut into circular specimens with a diameter of 3 mm using sterile biopsy punches to match the wound dimensions of the ex vivo wound models. Matrices were carefully inserted into the central wound defects after wound generation. Depending on the experimental group, wounds received either silk fibroin membranes, silk fibroin nonwoven matrices or remained untreated.

### 2.4. Bacterial Strains and Infection Model

*S. aureus* (DSM 799) and *P. aeruginosa* (DSM 19882; PA14) strains were obtained from the Leibniz Institute DSMZ (German Collection of Microorganisms and Cell Cultures, Braunschweig, Germany). Bacterial cultures were grown overnight in casein soy peptone broth (CSB) [15 g/L casein peptone (Carl Roth GmbH + Co. KG; Karlsruhe, Germany), 5 g/L soy peptone (Millipore, Darmstadt, Germany), 5 g/L NaCl] at 37 °C with shaking, then washed three times in 10 mL of phosphate-buffered saline (PBS; Sigma-Aldrich; St. Louis, MO, USA). Wounds were inoculated with 10 µL bacterial suspension containing 10^4^ colony-forming units (CFU) of either *S. aureus* or *P. aeruginosa* directly to the wound area.

### 2.5. Histology and Staining

Wound models were harvested on days 0, 5, 10 and 15 and fixed in 4% buffered Formafix (Grimm med. Logistik GmbH; Torgelow, Germany) for 3 days prior to dehydration and paraffin embedding. For all histological and immunofluorescence analyses, 3 µm-thick paraffin sections were obtained from the central wound region. Hematoxylin and eosin (H&E) staining was performed to assess tissue morphology, re-epithelialization and matrix integrity. Gram staining to visualize bacteria was performed using a Gram-staining kit (Carl Roth GmbH + Co. KG; Karlsruhe, Germany) according to the manufacturer’s instructions. Ki67 immunofluorescence staining was performed to assess cellular proliferation. Following deparaffinization and rehydration, antigen retrieval was carried out in 10 mM citrate buffer (pH 6.0) at 95 °C for 22 min. Sections were incubated overnight at 4 °C with monoclonal mouse anti-human Ki67 antibody (clone MIB-1, Dako Cytomation, Agilent Technologies; Glostrup, Denmark; 1:50 dilution), followed by incubation with Alexa Fluor^®^ 488-conjugated goat anti-mouse IgG secondary antibody (Invitrogen, Thermo Fisher Scientific; Waltham, MA, USA; 1:500 dilution) for 90 min at room temperature in the dark. Nuclei were counterstained with DAPI (Carl Roth GmbH + Co. KG; Karlsruhe, Germany) and mounted using Fluoromount-G™ mounting medium (Invitrogen, Thermo Fisher Scientific; Waltham, MA, USA). Brightfield histological images were acquired using a NanoZoomer^®^ S60v2MD slide scanner (Hamamatsu Photonics, Hamamatsu, Japan), and fluorescence images were acquired using a BZ-X1000 microscope (Keyence Corporation, Osaka, Japan), both at 20× magnification.

### 2.6. In Vitro Matrix Degradation Assay

To assess matrix stability under direct microbial and antiseptic challenge, silk fibroin membranes and nonwoven matrices were incubated under planktonic exposure conditions with *S. aureus*, *P. aeruginosa*, bacterial culture supernatants or the antiseptic solutions octenidine dihydrochloride (Octenisept^®^, Schülke & Mayr GmbH, Norderstedt, Germany), polyhexamethylene biguanide (PHMB; Serasept^®^, Serag-Wiessner GmbH & Co. KG, Naila, Germany), hypochlorite solution (NaClO) (Granudacyn^®^, Mölnlycke Health Care AB, Gothenburg, Sweden) or CSB as a control. Circular matrix specimens (6 mm diameter) were incubated in 500 µL of the respective solution at 37 °C. Bacterial exposure experiments were initiated with a starting inoculum of 10^6^ CFU/mL for each strain. Matrices were maintained under the respective conditions for up to 15 days, with medium or treatment solutions exchanged every second day. This exposure regimen was selected to provide a standardized model of repeated antiseptic challenge over a treatment interval relevant to the management of infected and hard-to-heal wounds. Matrix durability was documented macroscopically at predetermined time points and assessed using a semi-quantitative persistence score ranging from 0 to 4. Detailed scoring criteria are provided in Section Image analysis and quantification. Scoring was performed independently for each time point based on macroscopic preservation of matrix structure. Representative images were acquired using an M60 stereomicroscope (Leica Microsystems, Wetzlar, Germany) equipped with a 10× objective. For in vitro durability assays, each condition was assessed using nine independently incubated matrix specimens.

### 2.7. Scanning Electron Microscopy

Native silk fibroin membranes and nonwoven matrices, as well as representative matrices recovered from the in vitro degradation assay, were examined by scanning electron microscopy (SEM). For SEM, samples were fixed in glutaraldehyde/PVP solution (20% polyvinylpyrrolidone, 0.52% sodium nitrate and 2.5% glutaraldehyde in 0.1 M cacodylate buffer) for 1 h at 4 °C. Following repeated washing steps in cacodylate buffer, samples were dehydrated through a graded isopropanol series and air-dried overnight. Samples were subsequently sputter-coated with gold (108auto, Cressington Scientific Instruments Ltd., Watford, UK) and examined using a Crossbeam 340 scanning electron microscope (Zeiss AG, Oberkochen, Germany) operated at an accelerating voltage of 5 kV.

### 2.8. Image Analysis and Quantification

Histological and immunofluorescence image analysis was performed using ImageJ software 1.54g (National Institutes of Health, Bethesda, MD, USA). Re-epithelialization was quantified in the central area of the inner punch by measuring the combined length of newly formed epithelial tongues extending from both wound margins relative to the initial wound width and reported as percentage re-epithelialization according to the following formula:

Re-epithelialization (%) = (Total epithelial tongue length)/(Initial wound width) × 100

Ki67-positive nuclei were manually counted within regions of interest (ROI) encompassing the entire regenerating epithelial compartment, including both newly formed epithelial tongues. ROIs were defined for each wound section based on the extent of epithelial advancement and corresponded to the areas used for re-epithelialization measurements. Matrix integrity and durability were assessed by a single blinded investigator using a blinded semi-quantitative scoring system ranging from 0 to 4. For histological analyses, scoring was based on preservation of matrix architecture, continuity, thinning, fragmentation and visible material loss in H&E-stained sections. For macroscopic in vitro degradation assays, scoring was based on the preservation of the overall matrix structure and on visible material loss. In both cases, scores were assigned as follows: 4, largely preserved and continuous matrix; 3, mild degradation with limited thinning, discontinuities or structural alterations; 2, moderate degradation with evident thinning, fragmentation, fissures or splintering; 1, severe degradation with major structural disruption and substantial material loss; and 0, complete matrix loss or absence of recoverable matrix material. Representative examples of each score category were defined prior to analysis and applied consistently across all experimental groups (Supplementary Figure S1). Membrane thickness was quantified on H&E-stained sections using ImageJ software. Measurements were performed at multiple representative locations within each membrane section, and mean thickness values were calculated for each sample. Histological and macroscopic image evaluations were performed in a blinded manner.

### 2.9. Statistical Analysis

Statistical analyses were performed using GraphPad Prism version 11.0.2 (GraphPad Software, San Diego, CA, USA). Data are presented as mean ± standard deviation (SD) from three independent biological replicates (three individual skin donors). For each biological replicate, values obtained from three technical replicates (three wound models per donor) were averaged prior to statistical analysis. Re-epithelialization, Ki67-positive cell counts, and membrane thickness measurements were analyzed using two-way analysis of variance (ANOVA) followed by Tukey’s multiple comparisons test. Semi-quantitative matrix integrity and durability scores were treated as ordinal variables and analyzed using the Kruskal–Wallis test followed by Dunn’s multiple comparisons test. Statistical significance was defined as *p* < 0.05 for all analyses.

### 2.10. Declaration of Generative AI Use

**Figure 1:**
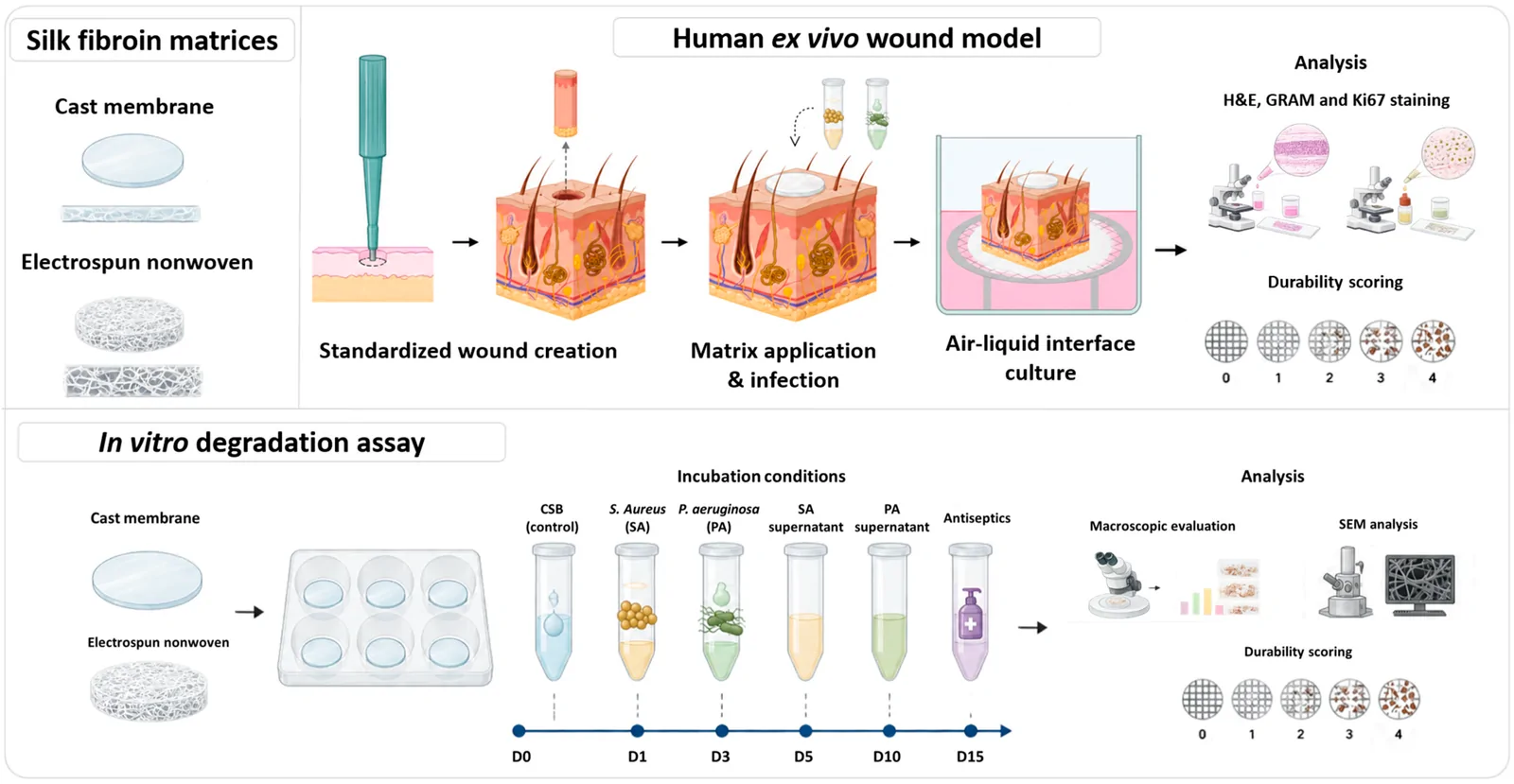
Experimental workflow. Silk fibroin membranes and electrospun nonwoven matrices were evaluated using two complementary experimental approaches. In the ex vivo arm, matrices were implanted into human skin wound models maintained under non-infected conditions or challenged with *S. aureus* or *P. aeruginosa*. Wound healing, tissue integration, bacterial colonization, cellular proliferation and matrix integrity were assessed by histological, immunofluorescence and microbiological analyses. In the parallel in vitro arm, matrices were incubated in bacterial cultures, bacterial supernatants, antiseptic solutions or control medium for up to 15 days. Matrix durability was evaluated by semi-quantitative persistence scoring and scanning electron microscopy (SEM).

Generative artificial intelligence (GenAI) tools were used during the preparation of this manuscript. Figure 1 (“Experimental workflow”) was created using ChatGPT GPT-5.5 (OpenAI; San Francisco, CA, USA) for conceptual development and visualization support and was created with BioRender (Toronto, ON, Canada; accessed 11 June 2026). The authors have reviewed and edited all AI-generated output and take full responsibility for the content of this publication. No GenAI tools were used for data collection, data analysis, interpretation of results, or generation of scientific conclusions.

## 3. Results

An overview of the experimental design and analytical workflow is shown in Figure 1. Two structurally distinct silk fibroin matrices, cast membranes and electrospun nonwoven matrices, were evaluated in non-infected and infection-driven human ex vivo wound environments as well as in complementary in vitro durability assays.

### 3.1. Structural Characterization of Silk Fibroin Matrices

**Figure 2:**
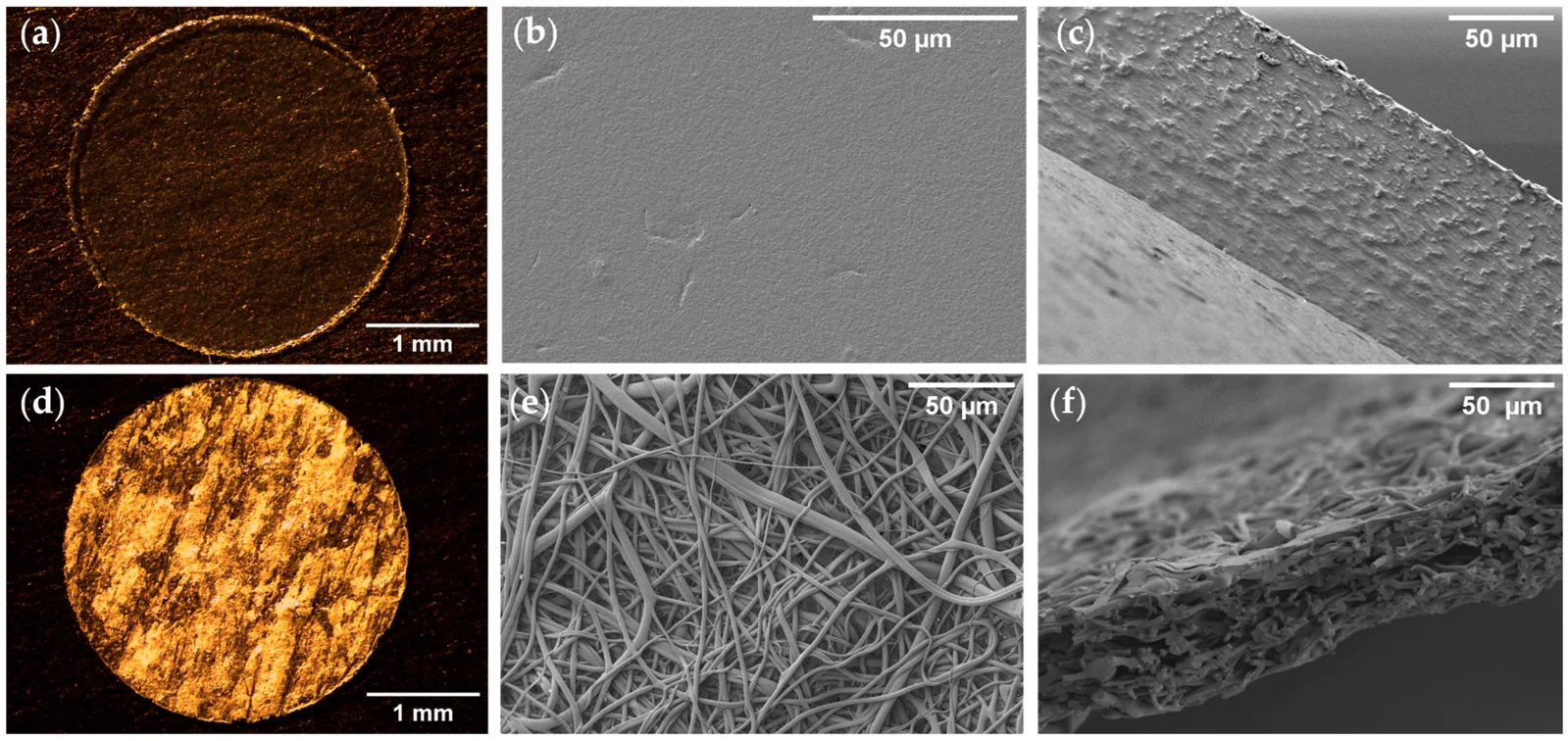
Structural characterization of silk fibroin matrices. Representative macroscopic and scanning electron microscopy images of silk fibroin membranes and electrospun nonwoven matrices. (**a**,**d**) Macroscopic appearance. (**b**,**e**) Surface morphology. (**c**,**f**) Cross-sectional morphology. Membranes exhibited a dense, compact architecture, whereas nonwoven matrices displayed an interconnected fibrous network with a porous three-dimensional structure. Scale bars: (**a**,**d**) 1 mm; (**b**,**c**,**e**,**f**) 50 µm.

The native silk fibroin membrane exhibited a dense, compact morphology, whereas the native nonwoven matrix displayed a highly porous fibrous architecture (Figure 2a–f). Macroscopically, membranes appeared smooth, homogeneous and translucent, whereas nonwoven matrices were opaque and textured (Figure 2a,d). Scanning electron microscopy confirmed these structural differences. Membranes exhibited a continuous, compact surface morphology and a dense cross-sectional architecture (Figure 2b,c). In contrast, nonwoven matrices consisted of an interconnected network of randomly oriented fibers and displayed a porous three-dimensional scaffold structure throughout the matrix thickness (Figure 2e,f).

### 3.2. Silk Fibroin Matrices Promote Regenerative Wound Healing Under Non-Infected Conditions

**Figure 3:**
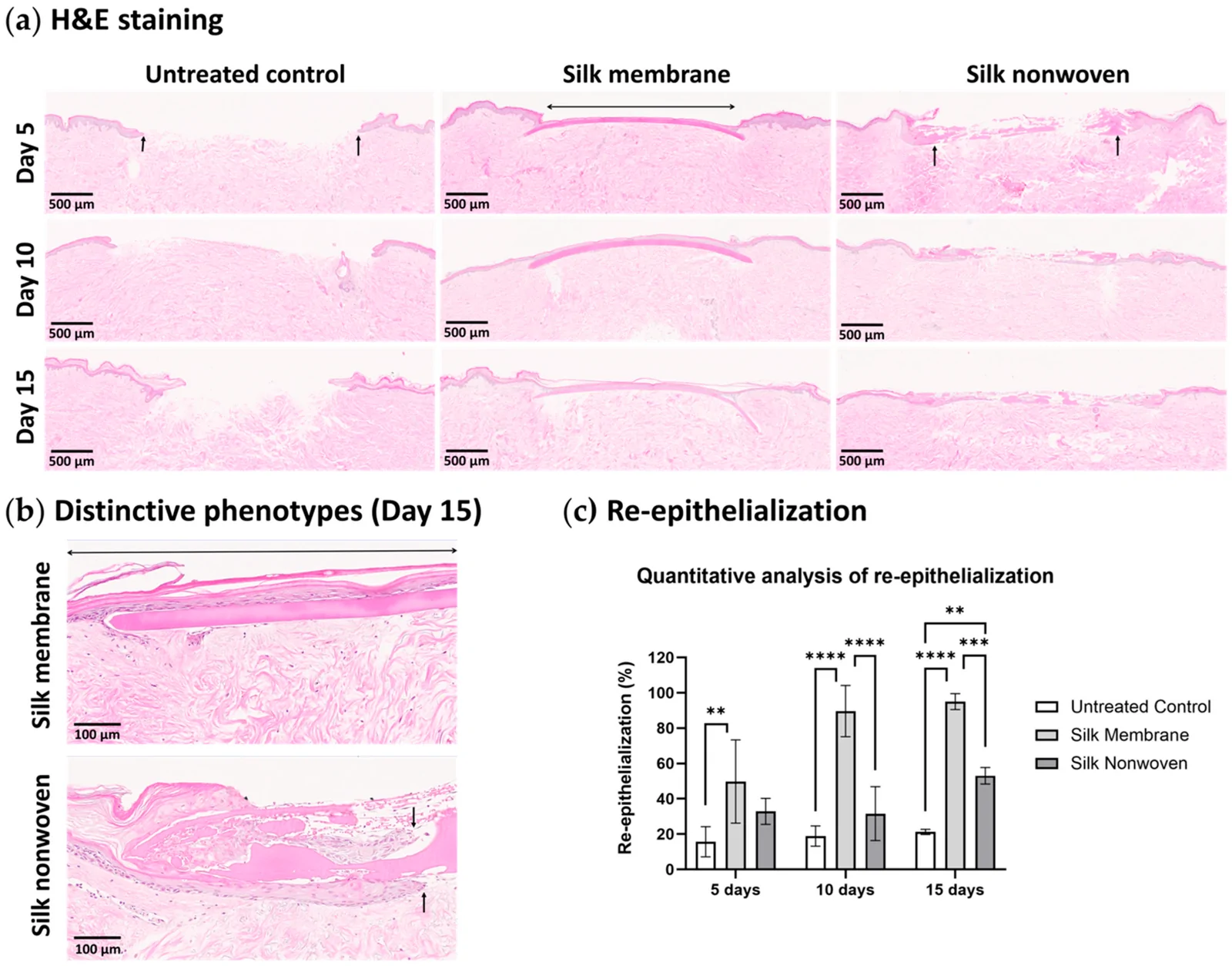
Regenerative effects of silk fibroin matrices under sterile ex vivo wound conditions. (**a**) Representative H&E-stained sections of untreated wounds and wounds treated with silk fibroin membranes or electrospun nonwoven matrices after 5, 10 and 15 days of cultivation. Scale bars = 500 µm. (**b**) High-magnification H&E images of silk fibroin membranes and nonwoven matrices illustrating increased cellularity in both matrix-treated groups and more pronounced tissue ingrowth in nonwoven-treated wounds. Scale bar = 100 µm. (**c**) Quantitative analysis of re-epithelialization expressed as the percentage of the initial wound width covered by epithelial tongues originating from both wound margins. Data are presented as mean ± SD from three independent biological replicates. Statistical significance is indicated as ** *p* < 0.01, *** *p* < 0.001 and **** *p* < 0.0001. Non-significant comparisons are omitted for clarity. Selected representative images are annotated to highlight the epithelial wound edges (single arrows) or the continuous neo-epithelial bridge (double-headed arrows).

Representative H&E-stained sections demonstrated progressive tissue regeneration in all groups over the 15-day cultivation period under non-infected conditions (Figure 3a). Untreated control wounds exhibited delayed epithelial advancement and persistent tissue defects, accompanied by a less organized tissue architecture. In contrast, wounds treated with silk fibroin membranes showed more extensive epithelial migration from the wound margins, resulting in re-epithelialization characterized by the formation of a continuous, multilayered stratified squamous epithelium across the wound surface. Nonwoven matrix-treated wounds likewise demonstrated cellular infiltration and integration of the scaffold into the surrounding tissue. Epithelial tongue formation was evident in both silk fibroin-treated groups; however, epithelial coverage appeared more complete in membrane-treated wounds, whereas nonwoven matrices were associated with more pronounced tissue ingrowth into the scaffold structure (Figure 3b). Membrane- and nonwoven-treated wounds additionally displayed a more regular tissue organization within the wound bed, characterized by increased cellularity and a denser distribution of spindle-shaped fibroblast-like cells (Figure 3b). A quantitative analysis of the measured wound edges confirmed histological findings of accelerated re-epithelialization (Figure 3c). After 5 days, membrane-treated wounds exhibited significantly greater re-epithelialization than untreated controls (*p* < 0.01), whereas no significant differences were observed for nonwoven matrices. By day 10, membrane-treated wounds showed significantly enhanced re-epithelialization compared with both controls and nonwoven matrices (both *p* < 0.0001). After 15 days, both silk fibroin-treated groups showed significantly higher re-epithelialization than controls (membrane: *p* < 0.0001; nonwoven: *p* < 0.01), whereas membrane-treated wounds exhibited significantly greater re-epithelialization than nonwoven-treated wounds (*p* < 0.0001). Overall, fibroin membrane treatment resulted in the most pronounced time-dependent enhancement of re-epithelialization throughout the observation period.

**Figure 4:**
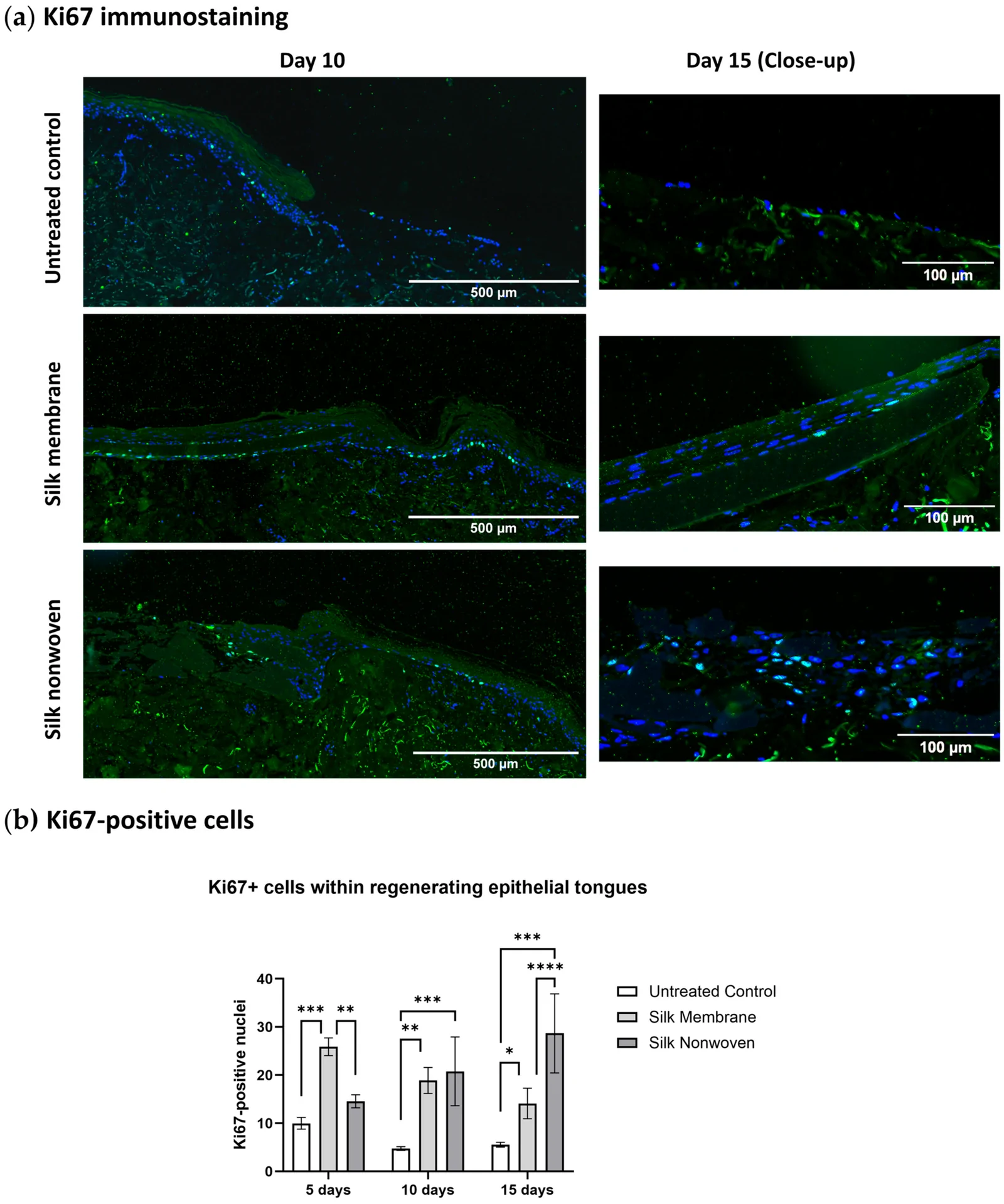
Proliferative activity during regeneration of non-infected ex vivo wounds treated with silk fibroin matrices. (**a**) Representative Ki67 immunostaining of untreated wounds and wounds treated with silk fibroin membranes or electrospun nonwoven matrices at days 10 and 15. Ki67-positive nuclei are shown in green and nuclei are counterstained with DAPI (blue). Scale bars = 500 µm (day 10) and 100 µm (day 15). (**b**) Quantification of Ki67-positive nuclei within regenerating epithelial regions. Data are presented as mean ± SD from three independent biological replicates. Statistical significance is indicated as * *p* < 0.05, ** *p* < 0.01, *** *p* < 0.001 and **** *p* < 0.0001. Non-significant comparisons are omitted for clarity.

Ki67 immunostaining revealed increased numbers of proliferating cells within the regenerating epithelial tongues of silk fibroin-treated wounds compared with untreated controls (Figure 4a). On day 15, nonwoven matrix-treated wounds exhibited numerous clusters of Ki67-positive cells distributed throughout the wound area and within the infiltrated nonwoven matrix. In contrast, Ki67-positive cells in control and membrane-treated wounds remained largely confined to the wound margins and regenerating epithelium. Quantification of Ki67-positive nuclei confirmed significant differences in proliferative activity between the experimental groups over time (Figure 4b). After 5 days, membrane-treated wounds exhibited significantly higher numbers of Ki67-positive nuclei than both untreated controls (*p* < 0.001) and nonwoven matrix-treated wounds (*p* < 0.01), whereas the nonwoven group did not differ significantly from controls. By day 10, both silk fibroin-treated groups showed significantly higher numbers of Ki67-positive nuclei than untreated controls (membrane: *p* < 0.01; nonwoven: *p* < 0.001), with no significant difference between membrane and nonwoven matrices. After 15 days, proliferative activity remained significantly elevated in both silk fibroin-treated groups relative to controls (membrane: *p* < 0.05; nonwoven: *p* < 0.0001). In addition, the nonwoven group exhibited significantly more Ki67-positive nuclei than the membrane group (*p* < 0.001).

### 3.3. Infection Fundamentally Alters Tissue Regeneration and Matrix Integrity

**Figure 5:**
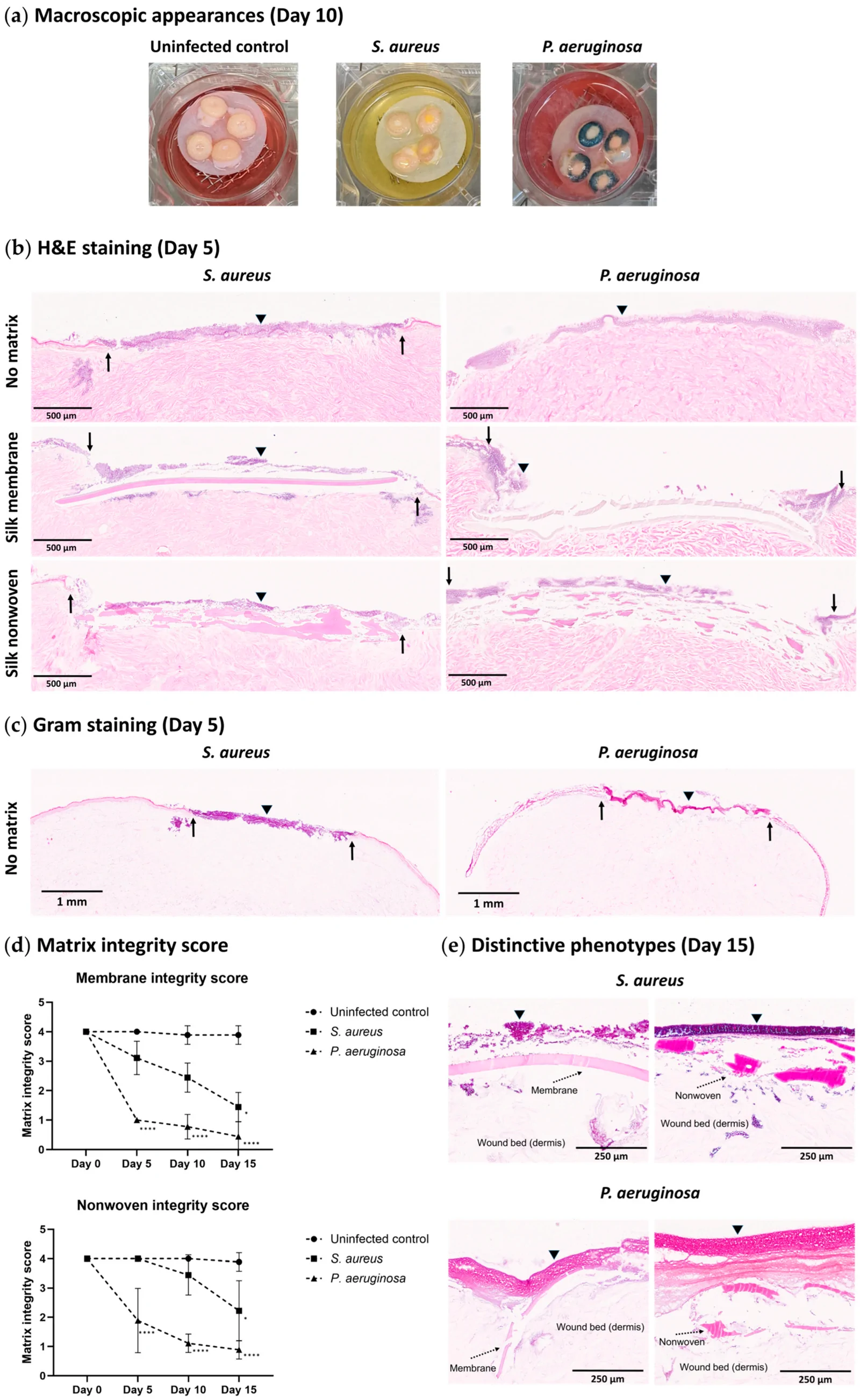
Infection fundamentally alters tissue regeneration and matrix integrity. (**a**) Representative macroscopic images of sterile, *S. aureus*- and *P. aeruginosa*-infected human ex vivo wound models at day 5. (**b**) Representative H&E-stained sections showing tissue morphology and re-epithelialization in infected wound models at day 5. Due to progressive tissue destruction and epidermal detachment, original wound margins became difficult to identify in infected wound models, particularly following *P. aeruginosa* infection. Scale bar = 500 µm. (**c**) Gram-staining illustrated bacterial localization and distribution in infected wound models on day 5. Scale bar = 1 mm. (**d**) Histological matrix integrity scores of silk fibroin membranes and nonwoven matrices under sterile conditions and following infection with *S. aureus* or *P. aeruginosa*. 0 = complete matrix loss; 4 = largely preserved matrix. Data are presented as mean ± SD of three independent donors. Asterisks indicate significant differences compared with the uninfected control at the corresponding time point. Non-significant comparisons are omitted for clarity (* *p* < 0.05 and **** *p* < 0.0001). (**e**) High-magnification H&E images of silk fibroin membranes and nonwoven matrices illustrating infection-associated matrix degradation. Scale bar = 250 µm. Representative annotations have been added to selected histological images. Solid arrows indicate the epithelial wound edges, arrowheads indicate the bacterial layer on the wound surface, and dotted arrows identify the silk fibroin membrane or electrospun nonwoven matrix.

Macroscopic examination revealed distinct pathogen-specific wound phenotypes (Figure 5a). *S. aureus*-infected wounds developed dense yellowish surface-associated bacterial accumulations that were initially concentrated within the wound center and remained associated with comparatively greater structural integrity of the wound model. In contrast, *P. aeruginosa*-infected wounds exhibited extensive green discoloration, abundant exudate, progressive epidermal detachment and markedly softer tissue consistency. These differences became increasingly pronounced during prolonged cultivation.

H&E staining revealed profound alterations in tissue architecture following infection with both *S. aureus* and *P. aeruginosa* compared with sterile controls (Figure 5b). Non-infected wounds exhibited progressive epithelial regeneration and preserved tissue organization, while infected wounds were characterized by extensive tissue destruction, widespread cellular loss and a complete absence of re-epithelialization, irrespective of matrix type. Furthermore, in both infection groups, viable skin cells were absent at later cultivation time points, leaving predominantly residual dermal extracellular matrix structures within the wound area. Consistent with the absence of detectable cellularity, no Ki67-positive cells were detected in infected wounds at day 5 irrespective of matrix type and bacterial species. Gram staining confirmed extensive bacterial colonization of infected wound models and revealed distinct spatial distribution patterns for the two pathogens (Figure 5c). On day 5, *S. aureus* was predominantly confined to the wound, with dense bacterial accumulations forming within the central wound. In contrast, *P. aeruginosa* rapidly spread beyond the wound margins and covered large portions of the tissue surface. This widespread colonization was accompanied by pronounced epidermal detachment at early time points. By day 10, *S. aureus* had expanded beyond the wound region and formed more extensive bacterial accumulations, whereas *P. aeruginosa* continued to form thick surface-associated bacterial layers covering large areas of the wound model and remained associated with extensive loss of epidermal integrity. Differences in matrix integrity were also apparent histologically (Figure 5d,e). Both *S. aureus* and *P. aeruginosa* infections were associated with progressive structural alterations of silk fibroin membranes and nonwoven matrices over time. Histological signs of degradation included matrix thinning, reduced staining intensity, fine fissures, structural discontinuities, fragmentation and partial loss of matrix material. These alterations were detectable in both infection groups but were substantially more pronounced in *P. aeruginosa*-infected wounds, where advanced matrix disintegration became evident at later cultivation time points. Representative high-magnification images illustrated these degradation patterns and revealed progressive fissure formation, fragmentation and loss of structural continuity in both matrix architectures, particularly following *P. aeruginosa* infection (Figure 5e). Matrix integrity scoring confirmed these observations and demonstrated significantly greater degradation in *P. aeruginosa*-infected wounds than in non-infected controls at all investigated time points for both matrix architectures (all *p* < 0.0001). In contrast, *S. aureus*-associated degradation progressed more gradually and reached statistical significance compared with sterile controls only on day 15 (*p* < 0.05). By the end of the cultivation period, both pathogens had caused substantial structural deterioration, although degradation remained consistently more pronounced in *P. aeruginosa*-infected wounds. Quantitative membrane thickness measurements further supported the histological assessment of matrix degradation (Supplementary Figure S2). Membrane thickness progressively decreased during cultivation under infected conditions and was significantly reduced in *P. aeruginosa*-infected wounds compared with non-infected controls from day 5 onwards. In contrast, thickness reductions following *S. aureus* infection were less pronounced and developed more gradually, consistent with the matrix integrity scores and histological observations.

### 3.4. Direct Bacterial Exposure Induces Pathogen-Dependent Degradation of Silk Fibroin Matrices In Vitro

**Figure 6:**
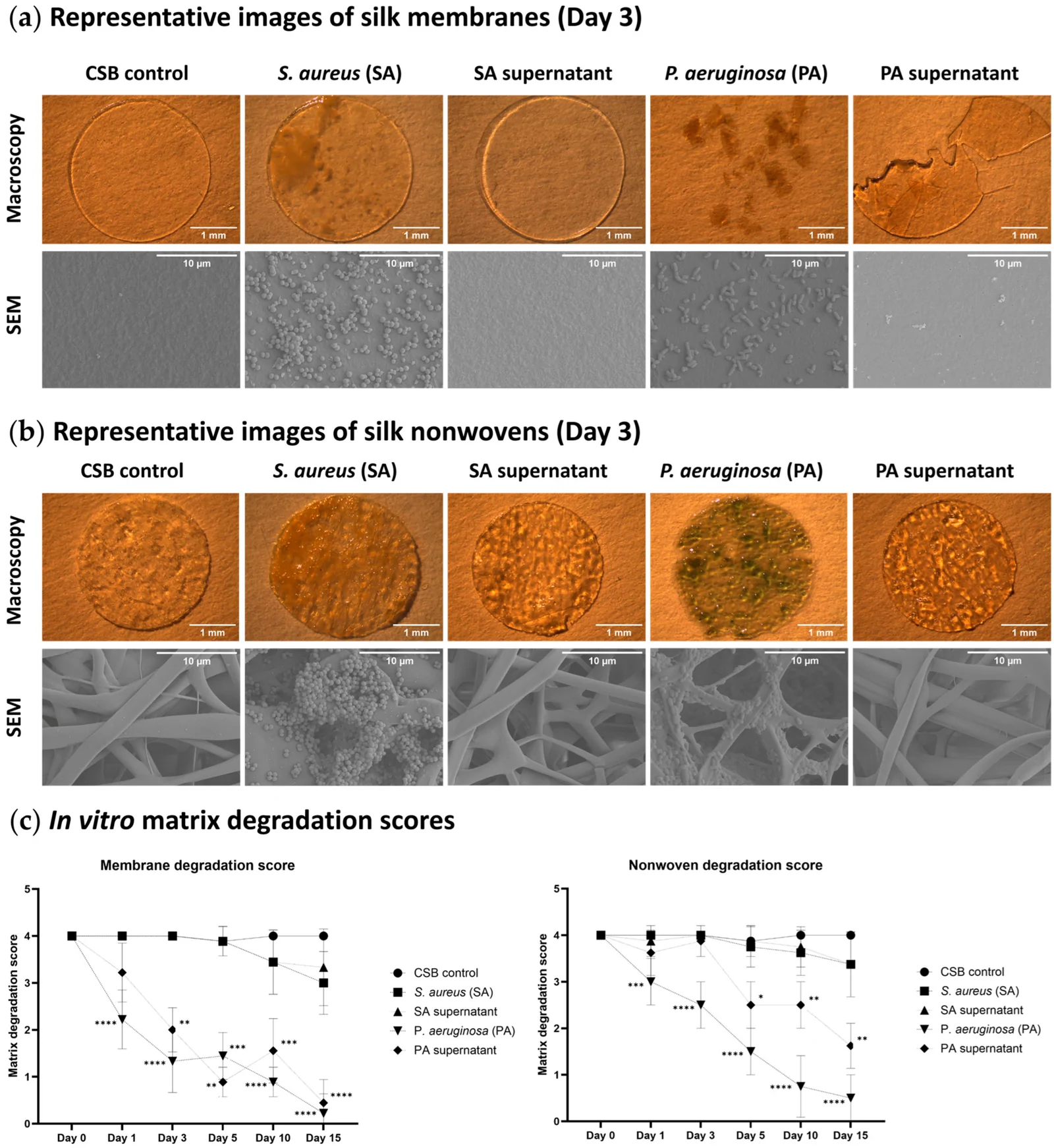
Direct bacterial exposure induces pathogen-dependent degradation of silk fibroin matrices in vitro. (**a**) Representative macroscopic and scanning electron microscopy images of silk fibroin membranes following 5 days of incubation in CSB, bacterial cultures or bacterial culture supernatants demonstrating pathogen-dependent structural attachment and alterations. (**b**) Representative macroscopic images of silk fibroin nonwoven matrices following 5 days of incubation under the indicated conditions. (**c**) Semi-quantitative durability scores of silk fibroin nonwoven matrices over the 15-day observation period. Asterisks indicate significant differences compared with the control at the corresponding time point. Non-significant comparisons are omitted for clarity (* *p* < 0.05, ** *p* < 0.01, *** *p* < 0.001 and **** *p* < 0.0001).

Macroscopic assessment of the in vitro degradation assay revealed condition-dependent differences in the stability of the silk fibroin matrix over a time period of 15 days (Figure 6). Both silk fibroin membranes and nonwoven matrices remained structurally stable throughout the observation period when incubated in CSB or exposed to octenidine dihydrochloride, PHMB or the hypochlorite solution, respectively. Hypochlorite exposure resulted in a brownish discoloration of both matrix types, but no visible loss of matrix integrity or persistence was observed. Exposure to *S. aureus* resulted in comparatively mild structural alterations. Membranes remained largely intact throughout the 15-day observation period, with minor fissures and localized discontinuities becoming apparent from day 10 onwards. Nonwoven matrices showed limited fiber loss and minor disruption of the scaffold architecture at later time points while largely retaining their overall structure. Comparable changes were observed following exposure to *S. aureus* culture supernatants. In both matrix types, extensive bacterial attachment and colonization were evident. In contrast, *P. aeruginosa* induced rapid and pronounced degradation of both matrix architectures. Membranes showed visible splintering and fragmentation as early as day 1, progressing to extensive structural disruption by day 5 and near-complete loss of recoverable material at later time points. Nonwoven matrices exhibited early fiber loss and fraying of scaffold margins, followed by progressive scaffold collapse and severe degradation or complete material loss by days 10–15. Exposure to *P. aeruginosa* culture supernatants produced similar but more gradual degradation patterns, with visible structural alterations becoming apparent from approximately day 5 onwards; representative pictures are shown in Figure 6a,b. Semi-quantitative durability scoring confirmed the macroscopic observations (Figure 6c). Both matrix architectures maintained high persistence scores after incubation in CSB and antiseptic solutions (Supplementary Figure S3). Exposure to *S. aureus* or its culture supernatants resulted in minor reductions in matrix persistence at later time points, consistent with the observed structural alterations, although these changes did not reach statistical significance relative to CSB controls. In contrast, exposure to *P. aeruginosa* bacterial cultures resulted in significant reductions in persistence scores for both silk fibroin membranes and nonwoven matrices from day 1 onwards (all *p* < 0.01). *P. aeruginosa* culture supernatants induced a more gradual decline in matrix persistence, reaching statistical significance from day 3 onwards in membranes and from day 5 onwards in nonwoven matrices. These differences persisted throughout the remainder of the observation period and were accompanied by progressive structural deterioration in both matrix architectures. No significant differences were observed between *P. aeruginosa* and corresponding supernatant-treated matrices at later time points, indicating that prolonged exposure to cell-free bacterial products was sufficient to reproduce substantial matrix degradation. Scanning electron microscopy further illustrated pathogen-dependent structural damage to silk fibroin matrices (Figure 6a,b). Native membranes displayed a compact, continuous surface morphology, whereas native nonwoven matrices exhibited an interconnected fibrous architecture. Following bacterial exposure, both matrix types exhibited surface erosion, fragmentation and loss of structural organization, with the most pronounced alterations observed after exposure to *P. aeruginosa*.

## 4. Discussion

This study investigated the regenerative performance and structural persistence of two silk fibroin-based wound dressing formats, a dense membrane and a nonwoven matrix, under both non-infected and bacterially infected wound conditions. In non-infected wound conditions, both matrices supported wound regeneration, as evidenced by enhanced re-epithelialization and increased proliferative activity compared with untreated controls. While the membrane promoted more rapid wound closure, the nonwoven matrix was associated with sustained cellular proliferation at later time points.

In contrast, bacterial infection substantially impaired these regenerative effects irrespective of matrix architecture. Moreover, matrix persistence was strongly pathogen-dependent, with *P. aeruginosa* causing substantially greater structural degradation than *S. aureus* in both ex vivo and in vitro models. Overall, these findings demonstrate that regenerative efficacy and material stability represent distinct biomaterial properties that are independently influenced by the wound microenvironment and microbial burden. These findings are consistent with previous reports demonstrating the favorable biocompatibility and regenerative potential of silk fibroin-based biomaterials in cutaneous wound healing [11,15]. Previous studies have shown that silk fibroin dressings may support keratinocyte migration, cellular proliferation and accelerated wound closure in both in vitro and in vivo wound models [13,14,15,23]. These observations are further supported by preclinical animal studies and a randomized controlled clinical trial demonstrating accelerated wound healing and favorable safety profiles for silk fibroin-based wound dressings [10,29]. Our findings extend these observations by directly comparing the regenerative behavior of two distinct silk fibroin architectures within the same human ex vivo wound model, thereby highlighting how matrix design influences the dynamics of tissue repair [30,31]. Despite these shared regenerative effects, distinct healing patterns were observed between the two matrix architectures: Silk fibroin membranes promoted more rapid re-epithelialization and greater wound closure, which may be related to their dense and continuous surface providing a more uniform interface for epithelial advancement [32]. However, this effect is likely influenced by multiple material properties, including matrix architecture, porosity, surface topography and mechanical stability, which collectively regulate cell–material interactions and can affect keratinocyte attachment, migration, proliferation and tissue regeneration [30,31]. In contrast, the highly porous nonwoven architecture more closely resembles the native extracellular matrix and was associated with sustained proliferative activity at later time points, consistent with previous in vitro studies [33,34]. This observation may indicate that nonwoven matrices have a greater capacity to support prolonged cellular infiltration and tissue remodeling, processes that could be particularly relevant in deeper wounds requiring substantial tissue regeneration. The persistence of Ki67-positive cell clusters within nonwoven-treated wounds suggests prolonged cellular infiltration and tissue remodeling, whereas membrane-treated wounds exhibited a gradual decline in proliferation as epithelial closure progressed, approaching levels observed in the surrounding healthy epidermis, consistent with the transition from the proliferative to the remodeling phase of wound healing [35]. Together, these findings indicate that matrix architecture influences not only the extent but also the temporal dynamics of tissue regeneration, with membranes favoring rapid epithelial coverage, while nonwoven matrices maintained proliferative activity over a longer period.

In contrast to the regenerative effects observed under non-infected wound conditions, bacterial infection profoundly altered the wound environment and was associated with a complete absence of re-epithelialization and proliferative activity irrespective of matrix architecture. These findings highlight the dominant influence of microbial burden on tissue repair and suggest that the regenerative benefits of biomaterials may be insufficient to overcome the detrimental effects of established wound infections. This observation is consistent with reports that the antibacterial activity of many silk-based biomaterials is primarily derived from incorporated antimicrobial agents rather than silk fibroin itself, suggesting limited protection against bacterial colonization and infection by native silk fibroin matrices [18,36]. These findings highlight the importance of complementing sterile biomaterial testing with infection-associated wound models, as bacterial infections may substantially alter both regenerative efficacy and biomaterial persistence. The bacterial inoculum was selected to enable reliable establishment of infection within the human ex vivo wound model while avoiding an excessively high initial bacterial burden, thereby providing a controlled infection challenge for biomaterial evaluation.

Chronic wound biofilms are known to impair healing through persistent inflammation, the production of cytotoxic metabolites and disruption of normal cellular repair processes, collectively creating a microenvironment unfavorable to regeneration [2,37]. To the best of our knowledge, this is the first standardized study comparing the biocompatibility and human tissue response to silk fibroin matrices under sterile conditions and in the presence of bacterial challenge. Interestingly, the effects of infection on matrix persistence were strongly pathogen-dependent. While *S. aureus* induced only moderate matrix degradation, exposure to *P. aeruginosa* resulted in pronounced structural deterioration in both the ex vivo wound model and the in vitro degradation assay. The observation that *P. aeruginosa* culture supernatants also promoted substantial matrix degradation suggests that secreted bacterial factors may contribute strongly to this process. In addition to extracellular proteases, *P. aeruginosa* secretes a diverse range of virulence-associated molecules, including redox-active metabolites, biosurfactants and quorum-sensing-regulated factors that can disrupt epithelial integrity, impair cellular migration and proliferation, and interfere with normal tissue repair processes [38,39]. Such factors may therefore contribute not only to reduced tissue regeneration but also indirectly to biomaterial deterioration through disruption of the local wound microenvironment. Among these secreted factors, proteolytic enzymes are particularly relevant because of their capacity to directly degrade proteinaceous substrates. *P. aeruginosa* produces a broad repertoire of extracellular proteases, including elastase and alkaline protease, which are capable of degrading host extracellular matrix components and may similarly contribute to the breakdown of protein-based biomaterials [40,41,42]. Notably, the ability of a *P. aeruginosa* LasB elastase-like protease to directly degrade fibroin-based silk from another lepidopteran (pine processionary caterpillar) suggests that similar proteolytic mechanisms may contribute to the deterioration of silk fibroin wound matrices under infected conditions [22]. Degradation of collagen-containing wound dressings and other biologically derived materials by bacterial proteases has also been reported previously, supporting the concept that microbial enzymatic activity represents an important but often underestimated determinant of biomaterial performance in infected wounds [5,7]. While the degradation observed in response to cell-free *P. aeruginosa* supernatants is consistent with a role for secreted proteolytic activity, the specific mediators responsible were not identified and additional extracellular factors may also contribute to the observed effects. In contrast, the comparatively limited degradation observed following *S. aureus* infection may reflect differences in the quantity and spectrum of secreted proteases, resulting in reduced matrix degradation under the conditions investigated [21,43]. The similarity between the ex vivo and in vitro findings further strengthens the biological relevance of these observations.

In both models, matrix degradation followed a similar pathogen-dependent pattern, with *P. aeruginosa* consistently exerting the strongest destructive effects. Together, these findings indicate that biomaterial persistence is not solely determined by intrinsic material properties but is also shaped by the surrounding microbial environment. The pronounced susceptibility of silk fibroin matrices to *P. aeruginosa* is particularly relevant in chronic wound infections, where this pathogen is frequently associated with delayed healing and poor clinical outcomes [44,45]. In contrast, the relatively limited effects observed for *S. aureus* indicate that not all wound pathogens pose the same risk to biomaterial stability. It is important to note that neither matrix architecture exhibited significant degradation after exposure to clinically relevant antiseptic solutions, suggesting that combining regenerative silk fibroin matrices with antimicrobial treatment strategies may be a promising approach for future wound care applications. However, whether combined matrix and antiseptic treatment can preserve regenerative function under infected wound conditions requires further investigation. Together, these findings highlight the importance of considering pathogen-specific interactions when evaluating regenerative biomaterials for clinical use.

Several limitations should be considered when interpreting these findings. Although the human ex vivo wound model preserves the native architecture and cellular composition of human skin, it does not fully recapitulate systemic host responses, including vascularization, immune cell recruitment and circulation-dependent antimicrobial defenses, nor the full complexity and temporal dynamics of chronic wound infections [27,46]. In addition, the study utilized tissue from three independent donors. The consistency of the observed effects across donors supports the robustness of the findings within the human ex vivo wound model. Nevertheless, future studies involving larger donor cohorts will be valuable to further evaluate the broader applicability of the results and account for potential inter-individual variability in wound healing responses. Furthermore, only mono-species infections of *S. aureus* and *P. aeruginosa* were investigated, whereas chronic wounds frequently harbor complex polymicrobial communities. Nevertheless, the combination of ex vivo wound infection models and complementary in vitro degradation assays provided a clinically relevant platform for assessing both regenerative performance and matrix persistence under controlled conditions.

Beyond the specific findings related to silk fibroin, the present study highlights a broader challenge in regenerative wound care. Biomaterials are frequently evaluated under sterile or simplified conditions that fail to reflect the complex biological and microbiological environments of chronic and infected wounds [47,48]. Relatively few studies have simultaneously investigated regenerative performance and biomaterial persistence under infected wound conditions, despite the likelihood that both parameters influence clinical outcomes [47,49,50]. Our findings demonstrate that regenerative efficacy and material persistence may diverge substantially under infection-driven conditions. These observations highlight the importance of incorporating infection-associated wound models into the preclinical evaluation of regenerative biomaterials. Ultimately, a more context-dependent assessment strategy may improve predictions of clinical performance and better support the development of wound therapies suited to the challenges of chronic and infected wounds.

## 5. Conclusions

This study demonstrates that the performance of regenerative wound biomaterials is strongly influenced by the wound environment. While silk fibroin matrices supported tissue regeneration under non-infected wound conditions, their behavior changed substantially in the presence of bacterial burden and infection, highlighting the importance of evaluating biomaterials under clinically relevant infection-associated conditions. Furthermore, the marked differences observed between *S. aureus* and *P. aeruginosa* emphasize that biomaterial persistence may depend not only on material properties but also on the microbial composition of the wound. Together, these findings support a more context-dependent approach to biomaterial development and evaluation, integrating both regenerative potential and infection-associated challenges. Such strategies may contribute to the development of more effective wound therapies for chronic and infected wounds.

## Data Availability

The data presented in this study are available from the corresponding author upon reasonable request. The data are not publicly available due to institutional ownership and data protection regulations of the University Medical Center Hamburg-Eppendorf (Hamburg, Germany).

## Supplementary Materials

The following supporting information can be downloaded at: https://www.mdpi.com/article/10.3390/biomimetics11080563/s1, Supplementary Figure S1: Representative histological examples illustrating the semiquantitative matrix integrity scoring system for silk fibroin membrane and electrospun nonwoven matrices. Supplementary Figure S2: Changes in the thickness of silk fibroin membranes following bacterial infection. Supplementary Figure S3: Effects of clinically used antiseptics and an antimicrobial solution on the integrity of the silk fibroin matrix.

## Abbreviations

The following abbreviations are used in this manuscript:

CSB	Casein soy peptone broth
DMEM	Dulbecco’s Modified Eagle Medium
H&E	Hematoxylin and eosin
NaClO	Hypochlorite solution
*P. aeruginosa*	*Pseudomonas aeruginosa*
PBS	Phosphate-buffered saline
PHMB	Polyhexamethylene biguanide
PTFE	Polytetrafluoroethylene
ROI	Regions of interest
*S. aureus*	*Staphylococcus aureus*
SD	Standard deviation
SEM	Scanning electron microscopy
