# Supplementary material for: Regenerative Performance and Structural Persistence of Silk Fibroin Matrices in Human Infected and Non-Infected Ex Vivo Wounds
Source: Biomimetics (Basel). 2026 Aug 6;11(8):563. doi: 10.3390/biomimetics11080563 (PMC13510087; doi:10.3390/biomimetics11080563)
Supplement: Supplementary file 1 [file biomimetics-11-00563-s001.zip › biomimetics-4437553-supplementary.pdf]

biomimetics

MDPI

Supplement

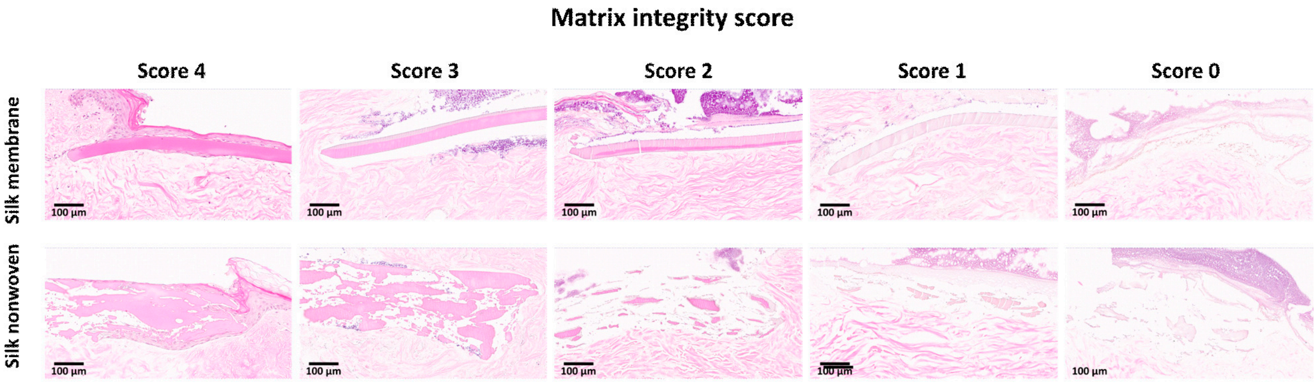

Supplementary Figure S1. Representative histological examples illustrating the semi-quantitative matrix integrity scoring system for silk fibroin membrane and electrospun nonwoven matrices. Matrix integrity was evaluated on H&E-stained sections by a single blinded evaluator using a five-point scoring system ranging from 4 to 0. Representative examples of each score are shown for the silk fibroin membrane (upper row) and electrospun nonwoven matrix (lower row). Score 4: largely preserved and continuous matrix. Score 3: mild degradation with limited thinning, discontinuities, or structural alterations. Score 2: moderate degradation with evident thinning, fragmentation, fissures, or splintering. Score 1: severe degradation with major structural disruption and substantial material loss. Score 0: complete matrix loss or no identifiable matrix remaining. Representative images were selected to illustrate the morphological criteria used for scoring and do not represent specific experimental conditions. Scale bars = 100 µm.

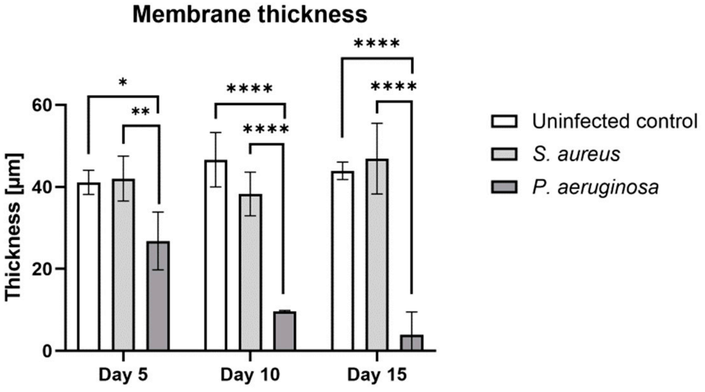

Supplementary Figure S2. Changes in the thickness of silk fibroin membranes following bacterial infection. Membrane thickness was quantified from histological sections after 5, 10 and 15 days of cultivation under sterile conditions or following infection with *S. aureus* or *P. aeruginosa*. Data are presented as mean ± SD from three independent donors. Statistical significance was determined using two-way ANOVA followed by multiple comparisons testing. *p < 0.05, **p < 0.01, ***p < 0.001 and ****p < 0.0001.

https://doi.org/10.3390/biomimetics11080563

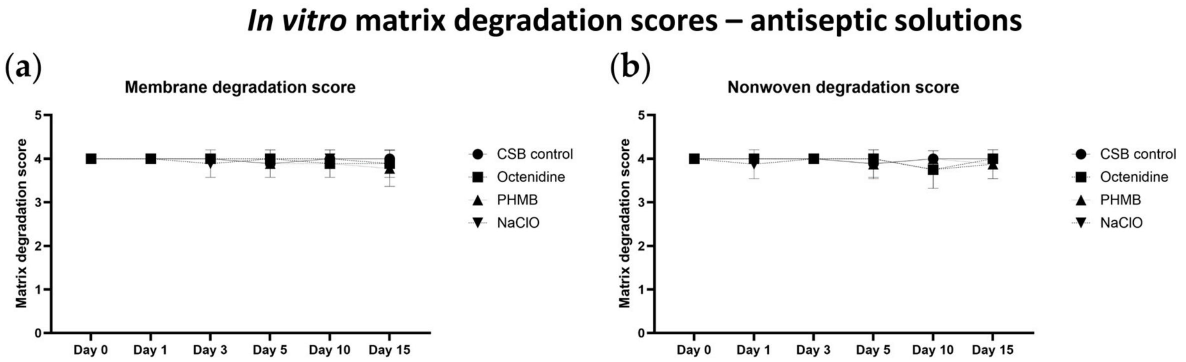

Supplementary Figure S3. Effects of clinically used antiseptics and an antimicrobial solution on the integrity of the silk fibroin matrix. (a) Matrix degradation scores of silk fibroin membranes following repeated exposure to the antiseptics octenidine dihy-drochloride and polyhexamethylene biguanide (PHMB) or hypochlorite solution (NaClO) over 15 days. (b) Matrix degradation scores of electro-spun silk fibroin nonwoven matrices exposed to the same antiseptic agents. Untreated matrices served as controls (CSB). Matrix degradation was assessed using a semiquantitative scoring sys-tem ranging from 0 (complete degradation) to 4 (intact structure). Data are presented as mean ± SD from nine in-dependent experiments. No significant differences in matrix integrity were observed be-tween antiseptic-treated and untreated matrices throughout the observation period.

https://doi.org/10.3390/biomimetics11080563
